# Development and Validation of a Clinical Scoring System for Predicting Risk of HCC in Asymptomatic Individuals Seropositive for Anti-HCV Antibodies

**DOI:** 10.1371/journal.pone.0094760

**Published:** 2014-05-06

**Authors:** Mei-Hsuan Lee, Sheng-Nan Lu, Yong Yuan, Hwai-I Yang, Chin-Lan Jen, San-Lin You, Li-Yu Wang, Gilbert L'Italien, Chien-Jen Chen, for the R.E.V.E.A.L.-HCV Study Group

**Affiliations:** 1 Institute of Clinical Medicine, National Yang-Ming University, Taipei, Taiwan; 2 Genomics Research Center, Academia Sinica, Taipei, Taiwan; 3 Division of Hepatogastroenterology, Department of Internal Medicine, Kaohsiung Chang Gung Memorial Hospital and Chang Gung University, Kaohsiung, Taiwan; 4 Molecular and Genomic Epidemiology Center, China Medical University Hospital, Taichung, Taiwan; 5 Graduate Institute of Clinical Medical Science, China Medical University, Taichung, Taiwan; 6 Department of Medicine, Mackay Medical College, New Taipei City, Taiwan; 7 Global Health Economics and Outcomes Research, Bristol Myers-Squibb, Princeton, New Jersey, United States of America; 8 Yale University School of Medicine, New Haven, Connecticut, United States of America; 9 Graduate Institute of Epidemiology and Preventive Medicine, College of Public Health, National Taiwan University, Taipei, Taiwan; University of Modena & Reggio Emilia, Italy

## Abstract

**Background:**

The development of a risk assessment tool for long-term hepatocellular carcinoma risk would be helpful in identifying high-risk patients and providing information of clinical consultation.

**Methods:**

The model derivation and validation cohorts consisted of 975 and 572 anti-HCV seropositives, respectively. The model included age, alanine aminotransferase (ALT), the ratio of aspirate aminotransferase to ALT, serum HCV RNA levels and cirrhosis status and HCV genotype. Two risk prediction models were developed: one was for all-anti-HCV seropositives, and the other was for anti-HCV seropositives with detectable HCV RNA. The Cox's proportional hazards models were utilized to estimate regression coefficients of HCC risk predictors to derive risk scores. The cumulative HCC risks in the validation cohort were estimated by Kaplan-Meier methods. The area under receiver operating curve (AUROC) was used to evaluate the performance of the risk models.

**Results:**

All predictors were significantly associated with HCC. The summary risk scores of two models derived from the derivation cohort had predictability of HCC risk in the validation cohort. The summary risk score of the two risk prediction models clearly divided the validation cohort into three groups (p<0.001). The AUROC for predicting 5-year HCC risk in the validation cohort was satisfactory for the two models, with 0.73 and 0.70, respectively.

**Conclusion:**

Scoring systems for predicting HCC risk of HCV-infected patients had good validity and discrimination capability, which may triage patients for alternative management strategies.

## Introduction

Hepatitis C virus (HCV) affects approximately 130–210 million people worldwide, and it is one of the leading causes of chronic hepatitis, cirrhosis, and liver cancer [1], [2]. Among patients chronically infected with HCV for 20–30 years, cirrhosis occurs in 20–30% [3]. Hepatocellular carcinoma develops in 1–4% of cirrhotic patients per year [4]. As a result of the successful hepatitis B virus vaccination program, HCV-related health burdens are emerging quickly in Asian countries [5].

Current US and European guidelines recommend screening for a history of risk of exposures to HCV and testing high-risk individuals who have identifiable risk factors [6], [7], [8]. However, fewer than half of those infected with HCV are aware of their infection [9], [10] and they may play as the infection sources in the community. Recent decision analysis showed that broader screening for HCV would be cost effective [11] and to expand HCV screening to general population over the current practice of only screening high-risk individuals is advocated [12]. Thus, it should be important to develop risk assessment tool for the individuals who have been identified to be seropositive of HCV after the implementation of new strategies of screening.

Several algorithms based on serum biomarkers have been developed recently that have included combinations of serum biomarkers to assist in the diagnosis of advanced liver disease [13], [14], [15], [16], [17], [18]. However, these algorithms have not yet been validated for their ability to predict the risk of end-stage liver diseases before onset. In addition, these algorithms have not focused on hepatocellular carcinoma.

A simple-to-use risk prediction models for liver disease progression are useful for improving patient care and disease stratification. In this study, we developed a noninvasive risk score system for hepatocellular carcinoma by integrating routinely measured clinical parameters among hepatitis C patients who were part of the Risk Evaluation of Viral Load Elevation and Associated Liver Disease/Cancer in HCV (R.E.V.E.A.L.-HCV) cohort. In addition, we applied the risk score system to an external cohort consisting of participants residing in an HCV-endemic area to validate its predictability.

## Materials and Methods

### Study population

#### R.E.V.E.A.L.-HCV Cohort for Risk Prediction Model Derivation

The R.E.V.E.A.L.-HCV cohort is derived from a community-based study which has been described previously [19], [20], [21]. In brief, participants living in seven townships in Taiwan provided written informed consent for interview, health examination, and blood collection during 1991–1992. Blood samples were obtained from each participant at study entry. In total, there were 1095 adults aged between 30–65 years old seropositive for antibodies against HCV (anti-HCV) but seronegative for hepatitis B surface antigen (HBsAg). They were followed till the end of 2008 for the incidence of hepatocellular carcinoma. The study protocol was approved by the institutional review board of the College of Public Health, National Taiwan University in Taipei.

#### High Risk Cohort for Risk Prediction Model Validation

Another cohort enrolled for the model validation included residents in southern Taiwan. The townships where the participants resided were endemic areas of HCV infection with high hepatocellular carcinoma mortality rates. The participants were invited to attend a community-based screening program in 2004–2005, and each participant provided informed written consent. The detailed enrollment procedures and characteristics of participants have been described previously [22], [23], . In total, we selected 572 anti-HCV seropositives who were seronegative for HBsAg and aged between 30–65 years old in the validation cohort; the participants in this validation cohort were followed till the end of 2008.

### Laboratory Examinations

The samples collected at study entry in both cohorts were tested for the seromarkers as followed. Tests on HBsAg, serum alanine aminotransferase (ALT) and aspartate aminotransferase (AST) level and anti-HCV were performed using commercial kits followed by standard procedures. The baseline serum samples collected from the participants in model derivation and validation cohorts were stored at −70°C until they were assayed for serum HCV RNA levels and HCV genotype. The serum HCV RNA was examined by the COBAS TaqMan HCV test, v2.0 (Roche Diagnostics, Indianapolis, NJ, USA). Serum samples with detectable HCV RNA were examined for HCV genotypes by LightCycler based PCR and melting curve analysis [20], [25], in R.E.V.E.A.L.-HCV cohort or by direct sequencing in the validation cohort.

### Ascertainment of Newly Developed Hepatocellular Carcinoma

Newly-developed hepatocellular carcinoma cases were identified by follow-up health examination and computerized data linkage. The participants in both cohorts obtained ultrasonography examinations performed by board-certified gastroenterologists every 6–12 months during follow-up. Once hepatocellular carcinoma was suspected sonographically, the patients were referred for confirmation based on the criteria of 1) histopathology; 2) two imaging techniques (abdominal ultrasonography, angiogram, or computed tomography); or 3) one imaging technique plus a serum α-fetoprotein level of 400 ng/mL or greater [26]. In addition to active follow-up, we performed computerized data linkage with the National Cancer Registration and the National Death Certification profiles from January 1, 1991, through December 31, 2008 to identify the occurrence of hepatocellular carcinoma.

### Statistical Analysis

Descriptive statistics characterizing participants in model derivation and validation cohorts were estimated. Differences between the two cohorts were evaluated with independent t tests for continuous and chi-squared tests for categorical variables. The follow-up years of each participant was calculated from the baseline recruitment to the date of hepatocellular carcinoma diagnosis, the date of death, or the date of last follow-up, which came first.

Multivariable Cox regressions were used to estimate the hazard ratio for each parameter included in the prediction equation for hepatocellular carcinoma. The proportional hazards assumption was verified. Parameters with statistically significant (p<0.05) hazard ratios were included in the risk prediction model. The Cox's proportional hazard regression coefficients of each included parameter were converted into an integer risk score by rounding the quotient of dividing the regression coefficient by a single constant. The constant selected was the regression coefficient for 5-year increase in age, allowing the integer risk score for a 5-year increase in age to be one [27]. The predicted risks for hepatocellular carcinoma were estimated by the sum of risk scores by the equation: 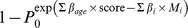, where P_0_ was the baseline disease free probability, *β_i_* was the regression coefficient for the ith variables (*X_i_*), and the *M_i_* denoted the mean level of *X_i_* [27].

To evaluate the predictive accuracy, the receiver operating characteristic (ROC) curve for each model was derived and the area under the ROC curve (AUROCs) was calculated. To evaluate the discriminatory ability of the risk models, the participants in both cohorts were classified into three groups by their sum risk scores, low, medium, high and the cumulative hepatocellular carcinoma risk of these three groups was estimated. The 25^th^ and 75^th^ percentiles of sum risk scores of patients affected with newly-developed hepatocellular carcinoma were used as the cutoff values in order to ensure that each group had an adequate number of hepatocellular carcinoma cases. The cumulative risk of hepatocellular carcinoma of participants with low, medium or high sum risk scores was estimated by Kaplan-Meier method, and the differences in cumulative hepatocellular carcinoma risk were compared by the log-rank test. All of the statistical analyses were performed by SAS version 9.1 (SAS Institute, Cary, NC).

## Results

**Figure 1:**
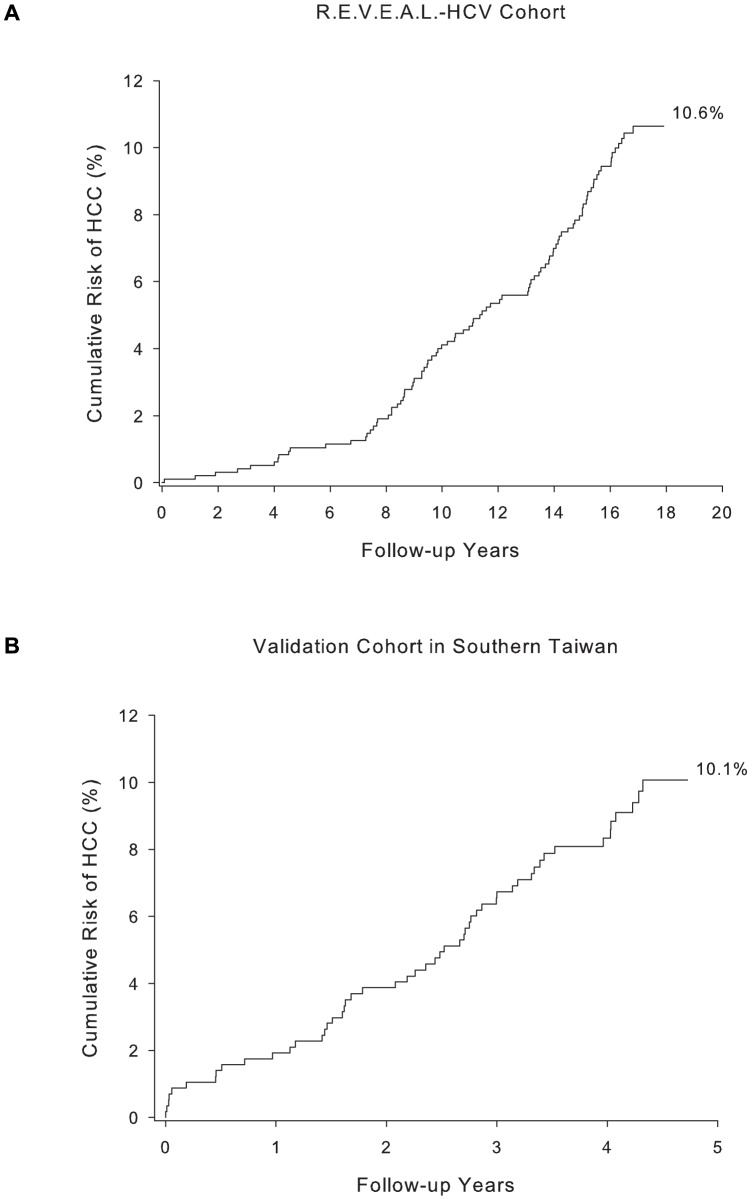
Cumulative risk of hepatocellular carcinoma after follow-up in (A) R.E.V.E.A.L.-HCV cohort and (B) high risk validation cohort in southern Taiwan.

The baseline characteristics of participants in the model derivation and validation cohorts were compared in Table 1. There were significant differences in the baseline characteristics of the two cohorts. The validation cohort had a significantly higher proportion of participants with older age, elevated serum ALT (>45 U/L) levels, detectable serum HCV RNA level, and cirrhosis status at study entry. The R.E.V.E.A.L.-HCV cohort was followed longer than the validation cohort with a median follow-up of 16.7 years, compared with 4.3 years for the validation cohort. Among the 975 participants in the R.E.V.E.A.L.-HCV cohort, 91 cases of hepatocellular carcinoma occurred after 14,821 person-years of follow-up, giving an incidence of 614 per 100,000 person-years. On the other hand, there were 52 incident hepatocellular carcinoma cases among 572 participants in the validation cohort after 2,265 person-years of follow-up, and the estimated incidence of hepatocellular carcinoma was 2296 per 100,000 person-years. Figure 1 showed the cumulative risk of hepatocellular carcinoma for participants in R.E.V.E.A.L.-HCV cohort and validation cohort, the latter had a significantly higher risk than the former (p<0.001). The higher hepatocellular carcinoma risk in the validation cohort reflected its more severe profile of risk predictors at study enrolment than the derivation cohort.

**Table 1:** Baseline characteristics of study participants and the number of hepatocellular carcinoma cases in model derivation and validation cohorts.

Baseline Predictors	R.E.V.E.A.L.-HCV cohort		High risk validation cohort		P value‡
	Total (N = 975), n (%)	HCC cases (n = 91)	Total (N = 572), n (%)	HCC cases (n = 52)	
Age					
Mean±SD	50.9±9.3	55.1±6.6	58.7±5.4	60.41±4.6	<0.001
30–39	163 (16.7)	3	0 (0.0)	0	<0.001
40–49	217 (22.3)	11	44 (7.7)	2	
50–59	399 (40.9)	49	214 (37.4)	13	
60–65	196 (20.1)	28	314 (54.9)	37	
Sex					
Female	550 (56.4)	45	386 (67.5)	31	<0.001
Male	425 (43.6)	46	186 (32.5)	21	
Serum ALT Levels (U/L)					
≤15	429 (44.0)	19	42 (7.3)	0	<0.001
16–45	387 (39.7)	40	178 (31.1)	3	
>45	159 (16.3)	32	352 (61.5)	49	
AST/ALT ratio					
<1	340 (34.9)	32	161 (28.1)	13	<0.001
≥1	635 (65.1)	59	411 (71.9)	39	
Liver cirrhosis					
No	961 (98.6)	85	532 (93.0)	38	<0.001
Yes	14 (1.4)	6	40 (7.0)	14	
Serum HCV RNA levels*					
HCV RNA undetectable	298 (30.6)	5	65 (12.0)	2	<0.001
Low RNA levels	339 (34.8)	36	293 (53.9)	31	
High RNA levels	338 (34.7)	50	186 (34.2)	12	
HCV genotype†					
Genotype non-1	271 (29.5)	20	224 (49.9)	27	<0.001
Genotype 1	351 (38.2)	50	160 (35.6)	9	

Abbreviations: SD, standard deviation; AST, aspirate aminotransferase; ALT, alanine aminotransferase.

*2.3×10^4^ as the cut-off for low and high serum levels of HCV RNA.

† HCV genotype was available only for those with detectable serum HCV RNA levels.

‡ compared the differences in the baseline characteristics for the participants in R.E.V.E.A.L.-HCV cohort and validation cohort.

### Derivation of Risk Prediction Model for Hepatocellular Carcinoma

We developed two risk prediction models. One was for all anti-HCV seropositives (included HCV RNA seropositives and HCV RNA seronegatives); and the other one was confining to the anti-HCV seropositives with detectable HCV RNA. All risk predictors included in each risk prediction model were statistically significantly associated with hepatocellular carcinoma (p<0.05) in the Cox's proportional hazards regression analyses. The regression coefficients of predictors in the risk prediction models were converted into integer risk scores as shown in Table 2 and Table 3. Participants with advanced age, elevated serum ALT levels, AAR higher than or equal to 1, presence of liver cirrhosis, elevated serum HCV RNA levels, and HCV genotype 1 infection had an increased risk score. The sum risk scores ranged from 0 to 22 for the model among all anti-HCV seropositives (Table 2). On the other hand, the risk prediction model for anti-HCV seropositives with detectable HCV RNA had the sum risk scores ranged 0–18 (Table 3).

**Table 2:** Coefficients and risk points of each baseline predictor for all anti-HCV seropositives (N = 975).

Predictors	Beta coefficient	Point	P value
Age at recruitment, 5 years increment	0.33	1	<0.001
Serum ALT Levels (U/L)			
≤15	Reference	0	
16–45	0.47	1	0.12
>45	1.23	4	<0.001
AAR			
<1	Reference	0	
≥1	0.56	2	0.04
Liver cirrhosis/HCV RNA level/HCV Genotype			
Without LC/HCV RNA undetectable	Reference	0	
Without LC/Low RNA level/genotype non 1	1.41	4	0.01
Without LC/High RNA level/genotype non 1	1.31	4	0.02
Without LC/Low RNA level/genotype 1	1.73	5	<0.001
Without LC/High RNA level/genotype 1	2.05	6	<0.001
Liver cirrhosis	3.29	10	<0.001

**Table 3:** 5-, 10-, and 15-year predicted risk and 95% confidence interval for hepatocellular carcinoma among all anti-HCV seropositives (N = 975).

Sum of risk score	5-year predicted risk (95% CI), %	10-year predicted risk (95% CI), %	15-year predicted risk (95% CI), %
0	0.02 (0.01–0.04)	0.08 (0.04–0.12)	0.19 (0.11–0.26)
1	0.03 (0.01–0.06)	0.11 (0.06–0.16)	0.26 (0.16–0.36)
2	0.04 (0.01–0.08)	0.15 (0.08–0.22)	0.36 (0.22–0.49)
3	0.06 (0.02–0.11)	0.21 (0.11–0.31)	0.49 (0.30–0.68)
4	0.09 (0.02–0.15)	0.29 (0.15–0.43)	0.68 (0.42–0.94)
5	0.12 (0.03–0.20)	0.40 (0.21–0.59)	0.94 (0.58–1.31)
6	0.16 (0.05–0.28)	0.55 (0.29–0.81)	1.30 (0.80–1.80)
7	0.23 (0.06–0.39)	0.76 (0.40–1.13)	1.80 (1.11–2.49)
8	0.31 (0.09–0.54)	1.06 (0.55–1.56)	2.49 (1.53–3.43)
9	0.44 (0.12–0.75)	1.46 (0.77–2.15)	3.43 (2.12–4.72)
10	0.60 (0.17–1.04)	2.02 (1.06–2.97)	4.72 (2.92–6.48)
11	0.83 (0.23–1.43)	2.78 (1.47–4.09)	6.48 (4.03–8.87)
12	1.15 (0.32–1.98)	3.84 (2.03–5.61)	8.86 (5.53–12.07)
13	1.59 (0.44–2.73)	5.27 (2.80–7.69)	12.06 (7.58–16.32)
14	2.20 (0.61–3.76)	7.23 (3.85–10.49)	16.31 (10.34–21.87)
15	3.03 (0.85–5.17)	9.88 (5.29–14.23)	21.85 (14.04–28.96)
16	4.18 (1.18–7.09)	13.41 (7.26–19.16)	28.93 (18.90–37.72)
17	5.74 (1.62–9.68)	18.09 (9.91–25.52)	37.69 (25.19–48.11)
18	7.86 (2.24–13.16)	24.15 (13.46–33.51)	48.07 (33.10–59.69)
19	10.72 (3.09–17.75)	31.81 (18.15–43.18)	59.66 (42.70–71.60)
20	14.54 (4.26–23.71)	41.15 (24.23–54.03)	71.56 (53.76–82.51)
21	19.55 (5.85–31.26)	52.03 (31.91–66.20)	82.48 (65.65–91.06)
22	26.02 (8.01–40.51)	63.85 (41.27–77.74)	91.04 (77.24–96.48)

The 5-year, 10-year, and 15-year predicted hepatocellular carcinoma risk by sum risk scores for the two models are shown in Table 4 and Table 5. Participants with higher sum risk scores had greater predicted risks of hepatocellular carcinoma. The 5-year predicted hepatocellular carcinoma risk ranged 0.02–26.02%; the 10-year risk ranged 0.08–63.85%; and the 15-year risk ranged 0.19–91.04% for the prediction model among all anti-HCV seropositives (N = 975). Secondly, the predicted risk for 5-, 10-, and 15-year for anti-HCV seropositives with detectable RNA was 0.10–22.38%; 0.34–59.31%; and 0.81–88.32% in correspondingly.

**Table 4:** Coefficients and risk points of each baseline predictor for anti-HCV seropositives with detectable HCV RNA (N = 677).

Predictors	Beta coefficient	Point	P value
Age at recruitment, 5 years increment	0.31	1	<0.001
Serum ALT Levels (U/L)			
≤15	Reference	0	
16–45	0.41	1	0.19
>45	1.09	4	0.003
AAR			
<1	Reference	0	
≥1	0.58	2	0.04
Liver cirrhosis/HCV genotype/HCV RNA levels			
Without LC/genotype non 1	Reference	0	
Without LC/genotype 1/low RNA levels	0.34	1	0.29
Without LC/genotype 1/high RNA levels	0.75	2	0.01
Liver cirrhosis	1.97	6	<0.001

**Table 5:** 5-, 10-, and 15-year predicted risk and 95% confidence interval for hepatocellular carcinoma among anti-HCV seropositives with detectable HCV RNA (N = 677).

Sum of risk score	5-year predicted risk (95% CI), %	10-year predicted risk (95% CI), %	15-year predicted risk (95% CI), %
0	0.10 (0.03–0.14)	0.34 (0.19–0.42)	0.81 (0.55–0.91)
1	0.13 (0.04–0.19)	0.46 (0.27–0.57)	1.11 (0.75–1.24)
2	0.18 (0.05–0.26)	0.63 (0.36–0.77)	1.50 (1.03–1.69)
3	0.24 (0.07–0.36)	0.86 (0.49–1.05)	2.04 (1.40–2.29)
4	0.33 (0.09–0.49)	1.17 (0.67–1.42)	2.78 (1.90–3.11)
5	0.45 (0.13–0.66)	1.59 (0.91–1.94)	3.76 (2.58–4.22)
6	0.61 (0.17–0.90)	2.17 (1.24–2.63)	5.09 (3.50–5.71)
7	0.84 (0.23–1.23)	2.94 (1.69–3.57)	6.88 (4.73–7.69)
8	1.14 (0.32–1.67)	3.99 (2.29–4.83)	9.26 (6.40–10.34)
9	1.55 (0.43–2.26)	5.39 (3.11–6.52)	12.40 (8.61–13.82)
10	2.11 (0.59–3.07)	7.28 (4.22–8.79)	16.51 (11.55–18.35)
11	2.86 (0.80–4.17)	9.78 (5.70–11.78)	21.80 (15.41–24.14)
12	3.88 (1.09–5.63)	13.09 (7.69–15.70)	28.47 (20.39–31.37)
13	5.25 (1.48–7.60)	17.41 (10.34–20.77)	36.66 (26.71–40.14)
14	7.08 (2.02–10.21)	22.94 (13.82–27.19)	46.34 (34.53–50.31)
15	9.52 (2.74–13.65)	29.90 (18.34–35.10)	57.19 (43.86–61.45)
16	12.75 (3.71–18.13)	38.38 (24.13–44.53)	68.53 (54.47–72.72)
17	16.96 (5.02–23.87)	48.31 (31.37–55.21)	79.31 (65.78–82.97)
18	22.38 (6.78– 31.04)	59.31 (40.13–66.53)	88.32 (76.81–91.04)

### Validation of Risk Prediction Models for Hepatocellular Carcinoma

In the evaluation of predictive accuracy of the risk model, the AUROCs for predicting 5-, 10- and 15-year hepatocellular carcinoma risk in the derivation set were 0.75, 0.83, 0.83 for model with all anti-HCV seropositives. On the other hand, the AUROC was 0.65, 0.77 and 0.73 for predicting the 5-, 10-, and 15-year risk of hepatocellular carcinoma. They indicated the sum risk scores had satisfactory to high validity for the prediction of hepatocellular carcinoma risk. The AUROCs for predicting 5-year hepatocellular carcinoma risk in the validation set was 0.73 and 0.70 for the two models.

**Figure 2:**
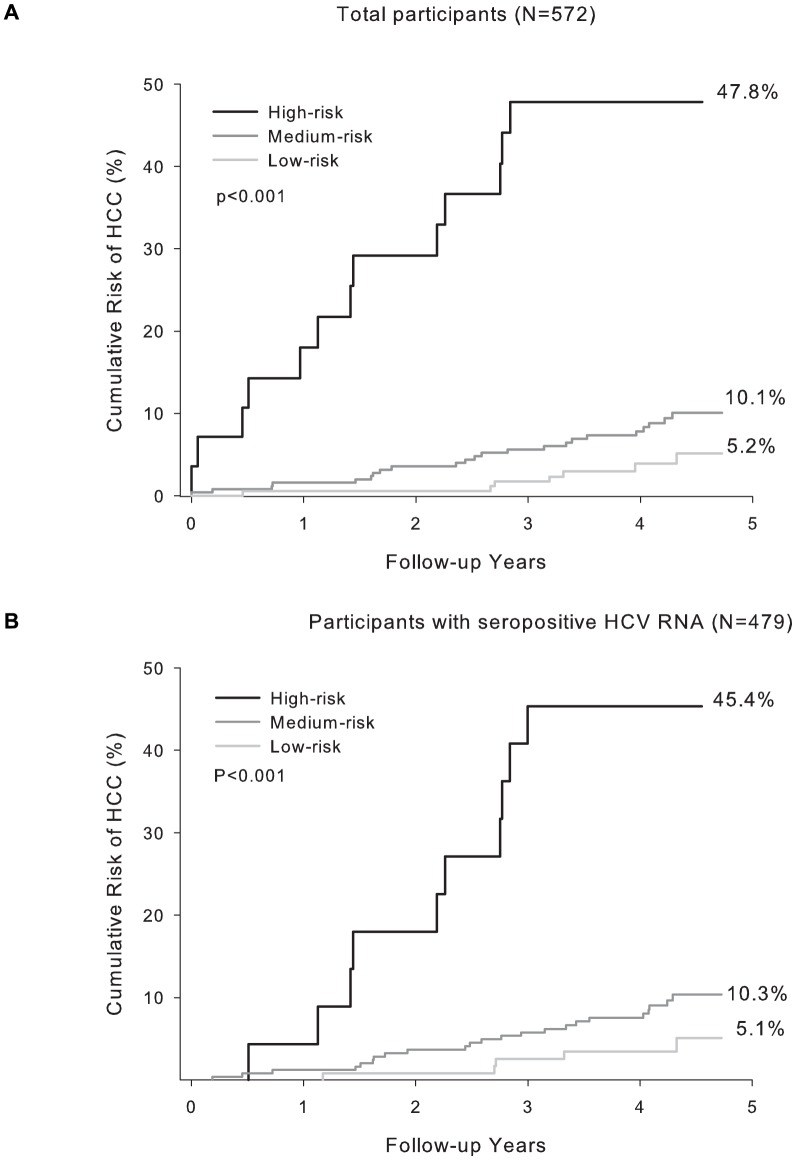
Cumulative risk of hepatocellular carcinoma of participants stratified by their sum of risk score in high risk validation cohort. (A) all anti-HCV seropositives (risk score <13 for low-risk, 13–18 for medium-risk, and ≥19 for high-risk group) (B) anti-HCV seropositives with detectable HCV RNA (risk score <9 for low-risk, 9–15 for medium-risk, and ≥15 for high-risk group).

In the evaluation of discriminatory ability of risk model in the validation set, participants with newly-developed hepatocellular carcinoma were found to have significantly higher sum risk scores than those unaffected (p<0.001) in each model. By applying to the model among all anti-HCV seropositives, the 25^th^ and 75^th^ percentile of the sum risk score among the anti-HCV seropositives affected with hepatocellular carcinoma in the validation set were 13 and 19. By using these values as cut-offs, the participants in the validation set were categorized by their sum risk scores into low, medium and high risk groups. The observed cumulative hepatocellular carcinoma risks of three groups are compared in Figure 2A. Secondly, by applying to the model confining to the RNA seropositives, the 25^th^ and 75^th^ percentile of the sum risk score among the anti-HCV seropositives with detectable HCV RNA was 15 and 19. By using the two cut-offs, the participants with detectable HCV RNA in the validation set could be differentiated into three groups and the cumulative risk of hepatocellular carcinoma of the three groups were depicted in Figure 2B. The cumulative risk curves for three predicted risk groups were all significantly different (p<0.001) in the two models.

## Discussion

This study used host (age, serum ALT level, ratio of AST to ALT, and cirrhosis status) and virus (serum HCV RNA and HCV genotype) factors to develop risk prediction models for hepatocellular carcinoma among chronic hepatitis C patients. During the natural course of liver disease progression, the serum levels of ALT, AST and HCV RNA may change dynamically. However, it may be more feasible and useful to provide risk prediction information to patients based on the measurement at the time of a clinic visit.

Chronic hepatitis C patients in Taiwan rarely received antiviral treatment due to its high cost and adverse effects until October 2003, when patients with abnormal serum ALT (>2×upper limit normal) and moderate fibrosis proven by liver biopsy (≧Metavir F2 or Ishak F3) could be reimbursed for treatment by the National Health Insurance. In the early beginning of antiviral treatment reimbursement, there remained few patients received standard care because the intrusive liver biopsy was not acceptable for the asymptomatic carriers. Moreover, the study areas (both the model derivation and validation cohorts) were relatively remote and the hepatological specialists were not popular in the townships. The participants in both cohorts were rarely treated due to the lack of health awareness and medical accessibility. However, the government recently modified the reimbursement criteria (serum ALT levels>1×upper limit normal and positive HCV RNA).

In order to successfully limiting HCV associated morbidity and mortality, to identify HCV infected persons and referral them for antiviral treatment is required [11], [28]. This risk prediction model derived from anti-HCV seropositives without treatment experience could be applied to the individuals who have been identified as anti-HCV seropositives in the community. In addition, the simple-to-use risk score system may help the anti-HCV seropositives estimate their long-term risk for hepatocellular carcinoma based on their own risk profiles. We developed two models. One was for anti-HCV seropositives which could be applied in general health center screening for HCV for lifestyle modifications and antiviral treatment considerations. The other model was for anti-HCV seropositives with detectable HCV RNA, which could be applied in clinical setting and enhancing patients' awareness and compliance for antiviral treatment. On the other hand, the model may provide health care practitioners communicate to anti-HCV seropositives for their future clinical management. Moreover, this risk prediction model will provide additional information for decision making in countries where financial resources are limited.

Community-based prospective studies of anti-HCV seropositives are rare. It is difficult to find an external cohort to validate risk prediction models developed from a derivation cohort. Fortunately, there was a screening-based follow-up cohort provided us a unique opportunity to examine the predictive performance of the prediction models derived from R.E.V.E.A.L.-HCV cohort. However, the follow-up years of the validation cohort were shorter than that of derivation cohort. In addition, participants in the validation cohort were older high-risk patients with thrombocytopenia or elevated serum α-fetoprotein level [22], [23], [24], [29]. Although this cohort had significantly different baseline characteristics compared with the R.E.V.E.A.L.-HCV cohort, the prediction accuracy was satisfactory. By using the risk score system, the participants in the validation cohort could be divided into three distinctive groups with low-, medium-, and high-risk of hepatocellular carcinoma. The findings imply the risk prediction models developed from derivation cohort have potential to be applied in the clinic to estimate the risk of hepatocellular carcinoma. Hepatitis C Antiviral Long-term Treatment against Cirrhosis (HALT-C) enrolled patients who have failed to achieve a sustained virologic response following antiviral therapy. All enrollees had detectable serum HCV RNA, presence of advanced hepatic fibrosis on liver biopsy (Ishak fibrosis score≥3), but no history of hepatic decompensation or hepatocellular carcinoma [13], [14], [16], [18]. The HALT-C study used the patient data to develop prediction models for histologically proven liver progression [13], [14], [16], [18]. The risk calculator for hepatocellular carcinoma was based on demographic variables (age and race), laboratory tests (alkaline phosphatase and platelet count), smoking history and presence of esophageal varices and is available on the website [16]. This algorithm provides a convenient way to calculate patients' hepatocellular carcinoma risk by entering their risk profiles, However, since only patients with advanced fibrosis were included, this may limit its generalizability. On the contrary, our study enrolled asymptomatic hepatitis C patients with external validation which provided an opportunity for clinicians to manage the disease before it entered the severe clinical stages.

The important finding of the role of IL28 polymorphisms in the prediction of the response to antiviral therapy has recently been reported [30], [31], [32]. The polymorphisms seem to be involved in the development of HCV-induced hepatocellular carcinoma and the course of HCV recurrence after liver transplantation [33]. It may be interesting to assess whether the prediction accuracy may increase by adding this genetic marker into hepatocellular carcinoma risk prediction models. However, the minor allele frequency of IL28 polymorphism was rare in Taiwanese population (0.04–0.06) [34], [35]. An enlarged sample size to obtain sufficient incident hepatocellular carcinoma cases through a collaborative multicenter study will be needed.

We did not include cigarette smoking and alcohol consumption into the models, because these two risk factors were not found to be significantly associated with hepatocellular carcinoma risk in our study. As the information of life-styles might not be available in clinical settings, the models without these two variables may be more applicable. In our study the clinical readily available seromarkers were included in the prediction models and the models could be widely utilized for consultations. Secondly, our risk prediction models did not include liver histology because the liver biopsy is not practical in these community-based studies. However, we included the AST to ALT ratio as a proxy of liver fibrosis status.

To our best knowledge, this study was the first one to develop and validate hepatocellular carcinoma risk models among asymptomatic hepatitis C carriers. The major limitation of this study was the validation cohort had a shorter follow-up period than that of the model derivation cohort. Thus, only the 5-year predicted risk for hepatocellular carcinoma could be validated. However, in our previous study we found that the serum levels of HCV RNA and ALT and HCV genotype were long-term predictors for hepatocellular carcinoma.[20] It was expected that the AUROC of the 10- and 15- year predicted risk for hepatocellular carcinoma should be improved in the validation cohort. The niche of our study was that the prediction models could be applied to relatively healthy patients with hepatitis C at early clinical stages. The predicted end-stage liver diseases could thus be prevented earlier. Individuals with high risk for hepatocellular carcinoma should be consulted for appropriate therapeutic management and intensively monitored to detect hepatocellular carcinoma at early stage.

Our scoring system models were developed from a long-term follow-up cohort with a moderate size. In addition, the models were validated in another sizable cohort and confirmed to have a satisfactory accuracy and discriminatory ability. Moreover, the parameters included are commonly recorded in clinics, which indicate that the scoring system could be used routinely in the clinic. The clinical practice guidelines indicate that all treatment-naïve patients with compensated disease and patients with fibrosis should be considered for therapy [36]. However, in patients with less severe disease, the indication for therapy should be individualized [36]. Our study enrolled asymptomatic chronic hepatitis C and estimated their risk profiles to provide useful information for the triage and clinical management of patients.

In conclusion, our risk prediction models combine readily available parameters in clinical practice and could be used to help physicians develop a disease management strategy. The prediction models had satisfactory discriminatory ability to differentiate patients into low, medium, and high risk for hepatocellular carcinoma and would be useful for planning therapeutic strategies and optimal utilization of health care resources.

### Disclaimers

All authors of this research paper have directly participated in the planning, execution, or analysis of the study.

All authors of this paper have read and approved the final version submitted.

The contents of this manuscript have not been copyrighted or published previously. The contents of this manuscript are not now under consideration for publication elsewhere.

The contents of this manuscript will not be copyrighted, submitted, or published elsewhere while acceptance by the Journal is under consideration.

There are no directly related manuscripts or abstracts, published or unpublished, by any author of this paper.
